# The association between adult-onset Still’s disease and collapsing glomerulopathy: a case report

**DOI:** 10.1186/s13256-022-03606-1

**Published:** 2022-10-14

**Authors:** Matas Orentas, Nilam Patel, Roger Rodby, Sobia Hassan

**Affiliations:** 1 Internal Medicine Department, Rush University Medical Center, Chicago, IL USA; 2 Nephrology Department, Rush University Medical Center, Chicago, IL USA; 3 Rheumatology Department, Rush University Medical Center, Chicago, IL USA

**Keywords:** Adult-onset Still’s disease, Collapsing glomerulopathy, Interferons

## Abstract

**Background:**

Collapsing glomerulopathy, characterized by marked hypertrophy and hyperplasia of the podocytes with eventual collapse of the glomerular tuft, is an important cause of end-stage renal disease. Among the many causes of collapsing glomerulopathy, autoimmune diseases, such as systemic lupus erythematosus, have been implicated. There are also rare reports of adult-onset Still’s disease, an autoinflammatory condition characterized by fever, rash, and inflammatory arthritis being associated with collapsing glomerulopathy.

**Case presentation:**

Herein, we present a review of three published cases, and present a new case of a 15-year-old African American female patient with collapsing glomerulopathy who was diagnosed with adult-onset Still’s disease 12 years later when she presented with fevers, arthralgias, sore throat, lymphadenopathy, hepatocellular injury, and elevated serum ferritin. Her collapsing glomerulopathy was initially well controlled following induction therapy with cyclosporine and prednisone and maintenance therapy with losartan. However, after developing adult-onset Still’s disease, she had multiple flare-ups despite various immunosuppressive therapies and developed worsening renal function, eventually progressing to end-stage renal disease.

**Conclusions:**

Our case-based review highlights a rare but important association between adult-onset Still’s disease and collapsing glomerulopathy, and postulates a possible pathophysiological link.

## Background

Adult-onset Still’s disease (AoSD) is a systemic condition most commonly presenting with quotidian fevers, rash, and inflammatory arthritis. It is also associated with specific organ dysfunction, particularly liver and cardiopulmonary disease [1]. Collapsing glomerulopathy (CG) is a variant of focal segmental glomerulosclerosis (FSGS) caused by a number of etiologies of podocyte injury, but specifically with epithelial cell hypertrophy and hyperplasia, and eventual collapse of the glomerular tuft leading to proteinuria, global glomerular sclerosis, and often end-stage renal disease (ESRD) [2, 3]. CG is a histologic pattern of injury that has been associated with viral infections, drugs, and autoimmune or autoinflammatory diseases such as systemic lupus erythematosus (SLE) and AoSD [4]. To highlight the rare association between AoSD and CG, we review three published cases and report an additional case of a 30-year-old female who was diagnosed with CG via renal biopsy in 2006 and then AoSD 12 years later when she developed fevers, arthralgias, neutrophilic leukocytosis, elevated liver function tests (LFTs) and ferritin, and serositis.

## Case presentation

**Fig. 1:**
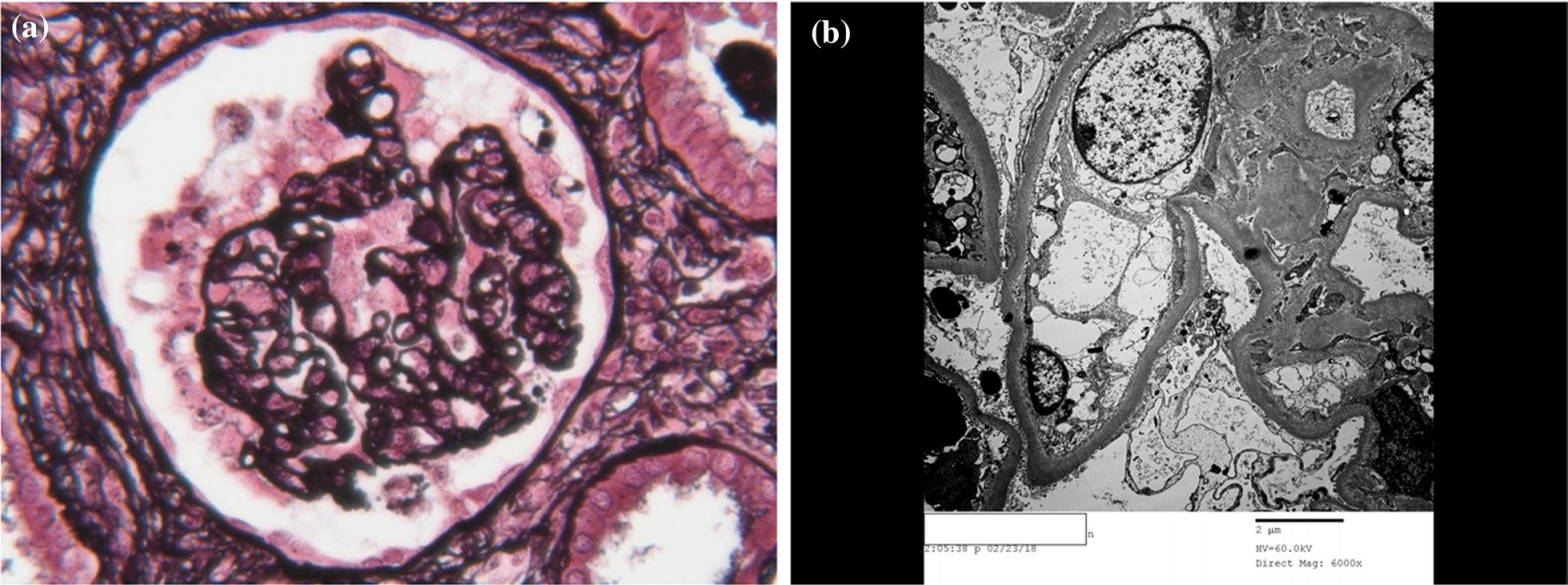
**a** A silver stain showing parietal epithelial cell proliferation and collapse of the glomerular tuft. **b** An electron micrograph showing diffuse foot process effacement

A 15-year-old African American female with a past medical history of allergic rhinitis and obesity presented with lower extremity and periorbital edema, and was found to have nephrotic range proteinuria, hypoalbuminemia, and renal insufficiency (Table 1). Her father had a history of ESRD of unknown etiology and died from a myocardial infarction in his forties. She worked at T Mobile as a salesperson. She had not traveled outside of Illinois, which is where she was born and had lived her whole life. She denied alcohol or tobacco use and her only medication was loratadine as needed for allergic rhinitis. She underwent a renal biopsy and nine out of the twenty sampled glomeruli featured segmental or global scarring. In the non-globally sclerotic glomeruli, the glomerular tufts showed marked visceral epithelial cell proliferation and the glomerulus showed global collapse. Diffuse foot process effacement was present on electron micrography (Fig. 1). Work-up for secondary causes of CG including human immunodeficiency virus (HIV) and parvovirus B19, was negative. She received oral prednisone and cyclosporine for about 2 years (dose unknown), achieved partial remission (defined as a reduction in proteinuria by 50% or more and stable renal function), and was maintained on oral losartan 100 mg daily.

**Table 1:** Patient characteristics at various timepoints of disease course

	Nephrotic syndrome presentation	Nephrotic syndrome remission	AoSD presentation	Rheumatology follow-up	Death
Age (years)	15	16–27	27	27–30	30
White blood count (4–10 K/μL)	5.07	2.5–11.05	18.74	3.2–14.18	30.39
Hemoglobin (12–16 g/dL)	13.2	11.9–14.8	9.7	9.8–14.9	7.7
Platelet count (150–399 K/μL)	296	169–320	478	143–293	121
Serum creatinine (0.65–1.00 mg/dL)	2.4	1.1	2.95	1.52–4.87	17.33
Serum albumin (3.5–5.0 g/dL)	1.9	3.9	1.4	2.3–3.9	1.4
Urine protein/creatinine ratio (0.021–0.161 g/g)	12.2	< 1	5.4	0.81–2.2	6.9
Urinalysis	Protein > 300, moderate blood, 5 WBC, 5 RBC, no casts	Protein 30, no blood, no WBC/RBC, no casts	Protein > 300, large blood, 10 WBC, 5 RBC, no casts	Protein 30, no blood, no WBC/RBC, no casts	Protein > 600, large blood, 1 WBC, 9 RBC, no casts
Renal biopsy	Collapsing glomerulopathy	Not applicable	Collapsing glomerulopathy	No further biopsies	No further biopsies
Fever	No	No	Yes	Intermittently	Yes
Arthralgias/arthritis	No	No	Yes	Intermittently	Yes
Salmon rash	No	No	No	No	No
Sore throat	No	No	Yes	Intermittently	No
Lymphadenopathy	No	No	Yes	No	Yes
Hepatomegaly/splenomegaly	No	No	No	No	Yes
AST/ALT/LDH elevation	No	No	Yes (364/173/695)	Intermittently	Yes (488/49/1310)
Ferritin (ng/mL)	Not assessed	Not assessed	> 40,000	148–2116	8749
ANA/RF serologies	ANA 1:80 speckled, RF < 20	No serologies	ANA 1:80 homogenous, RF 62	No serologies	ANA < 1:40, RF 16
Microbiology	HIV and parvovirus B19 negative	Not applicable	HIV, EBV, CMV, influenza, histoplasmosis, hepatitis B and C, RPR, and quantiferon gold negative	Not applicable	COVID-19, quantiferon gold, and C. diff toxin negative
Blood cultures	Not applicable	Not applicable	Blood culture ×3 negative	Not applicable	Blood culture ×2 negative

*K/uL* thousand per microliter,* g/dL* gram per deciliter,* mg/dL* milligram per deciliter,* gm/gm* gram per gram,* AST* aspartate transaminase,* ALT* alanine transaminase,* LDH* lactate dehydrogenase, *ng/ml* nanogram per deciliter,* WBC* white blood cell,* RBC* red blood cell,* ANA* antinuclear antibodies, *RF* rheumatoid factor,* HIV* human immunodeficiency virus,* EBV* Epstein-Barr virus,* CMV* cytomegalovirus,* RPR* rapid plasma reagin, * C. diff* Clostridioides difficile

**Fig. 2:**
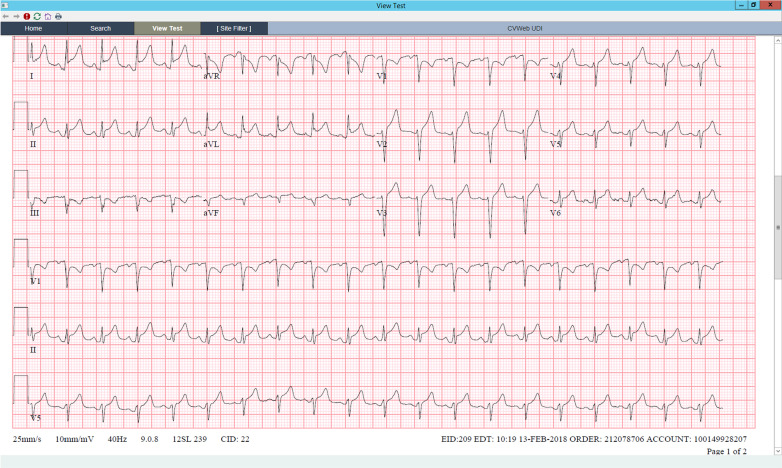
Electrocardiogram with diffuse PR segment depression and ST segment elevation consistent with pericarditis

**Fig.3:**
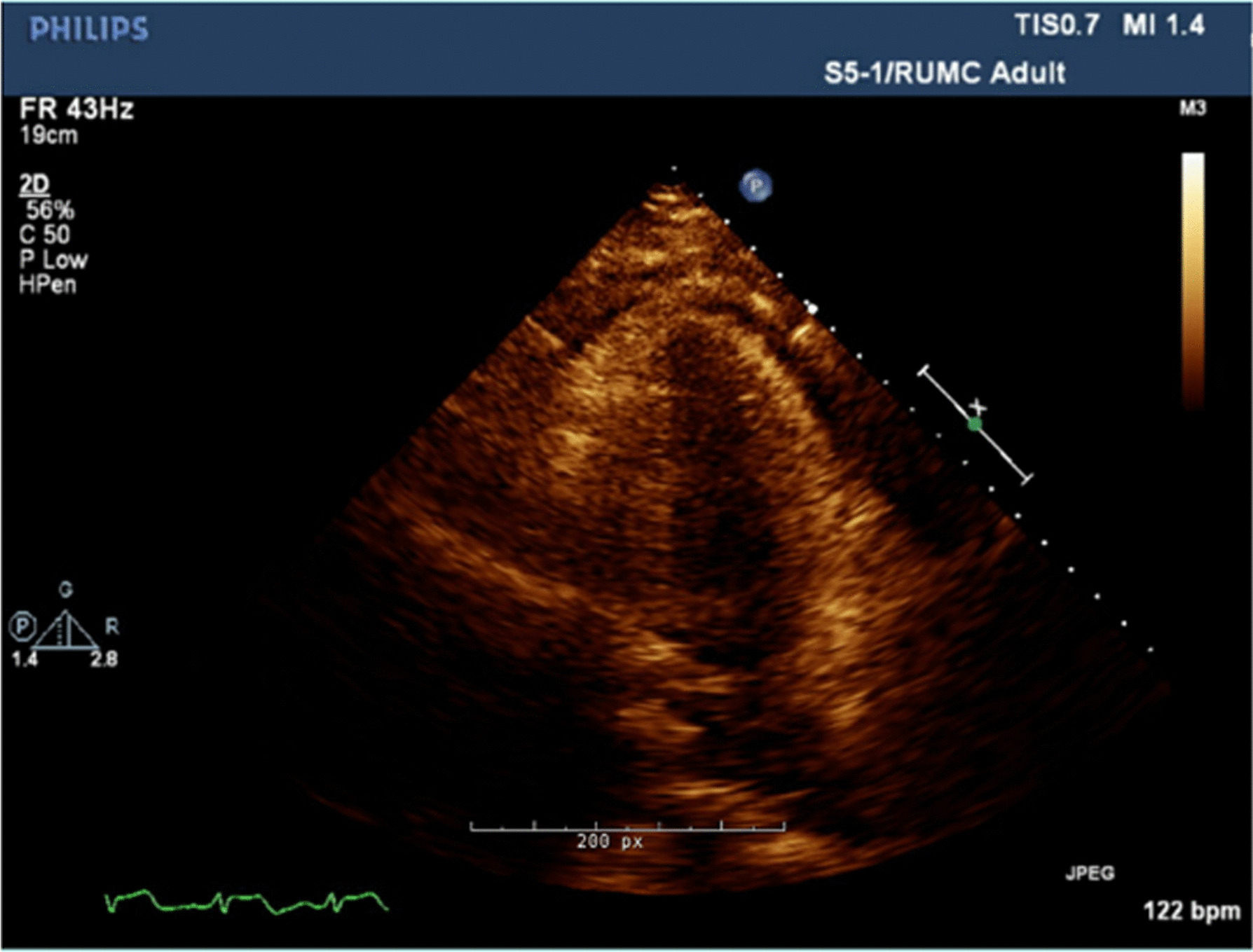
Transthoracic echocardiogram with mild pericardial effusion

**Fig. 4:**
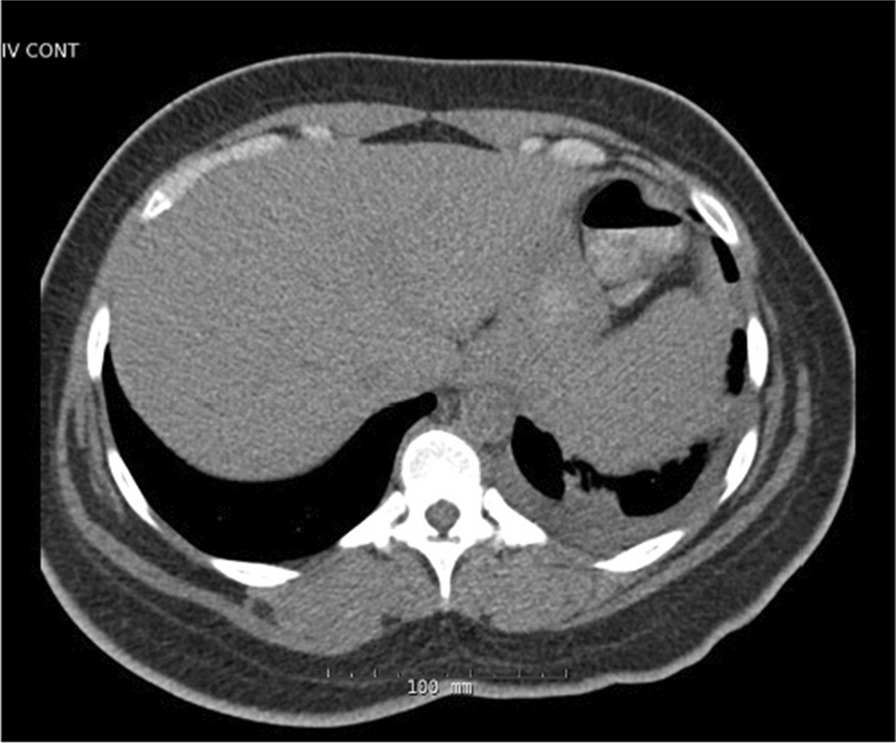
Computed tomography chest, abdomen, pelvis with small pleural effusion and atelectasis at left lung base

At the age of 27, she presented to the hospital with several weeks of fever, sore throat, chest pain, and body aches. Outpatient testing for influenza, streptococcus, and Epstein–Barr virus was negative. On admission, her temperature was 104.4 F, blood pressure 117/76 mmHg, and heart rate 114 beats per minute. Physical examination revealed a cooperative female in no acute distress, normal conjunctiva, unremarkable oropharynx without pharyngeal erythema, anterior cervical lymphadenopathy, sinus tachycardia with no additional heart sounds or murmurs, clear lung fields, benign abdomen without hepatosplenomegaly, diffuse joint tenderness but no joint swelling, no rash, normal gait, and intact cranial nerves, motor strength, sensation, and reflexes. Laboratory examination was significant for leukocytosis, acute on chronic kidney disease, nephrotic range proteinuria, and mild transaminitis. Serologies showed antinuclear antibodies (ANA) of 1:80, negative dsDNA, normal complement levels, rheumatoid factor (RF) of 60 IU/mL, negative antineutrophil cytoplasmic antibodies (ANCA), anti-Sjögren's syndrome type A (SSA) > 8, and negative anti-Sjögren's syndrome type B (SSB). Ferritin was > 40,000 ng/mL (Table 1). Electrocardiogram (EKG) was noted to have diffuse ST segment elevations (Fig. 2), troponin was 2 ng/mL, and transthoracic echocardiogram (TTE) showed mild pericardial effusion (Fig. 3); this constellation was consistent with pericarditis. Computed tomography (CT) of the chest, abdomen, pelvis (CAP) showed a small left sided pleural effusion (Fig. 4). CT of the neck showed enlarged bilateral lymph nodes without evidence of abscess.

After no underlying infectious or malignancy causes were identified, a primary rheumatological process was considered. She was given a trial of oral prednisone (60 mg daily), with resolution of fever and leukocytosis and symptomatic improvement. Oral colchicine (0.6 mg daily for 1 month) was added to treat the pericarditis. Repeat renal biopsy re-demonstrated CG and, specifically, absence of SLE features.

Her rheumatological presentation was felt to be most consistent with AoSD, given the fevers, arthralgias, leukocytosis, sore throat, lymphadenopathy, elevations in aspartate transaminase/alanine transaminase (AST/ALT), and the markedly elevated ferritin. Although she had a positive RF, her clinical presentation was not compatible with rheumatoid arthritis. Given her low titer ANA and positive SSA, the possibility of SLE was considered. She was maintained on oral hydroxychloroquine (400 mg daily) and monitored closely in the rheumatology clinic, but over years of follow-up her presentation remained more consistent with AoSD rather than SLE. Additionally, she lacked signs or symptoms to suggest a diagnosis of Sjogren’s disease.

During follow-up in the rheumatology clinic, attempts to wean oral prednisone below 10 mg daily were unsuccessful, resulting in flares characterized by fevers, sore throat, facial swelling, arthralgias, myalgias, urticarial rash, flank pain, chest pain, and associated elevated inflammatory markers. Oral methotrexate (15 mg weekly) was added in August 2018 as a steroid sparing agent, with initial improvement. Due to a recurrence of pericardial effusion and inability to increase her methotrexate dose due to chronic kidney disease (CKD), tocilizumab (8 mg/kg intravenous infusion every 4 weeks) was added in December 2018. Given continued disease activity, the patient was switched to subcutaneous anakinra (100 mg daily) in January 2020 and methotrexate and tocilizumab were discontinued. During this time, her CG remained stable on oral losartan (100 mg daily).

Her rheumatologic disease was quiescent for several months on anakinra, but then the patient developed fever and dry cough requiring admission in March 2020. Infectious work up was negative. Laboratory work-up was concerning for flare of her AoSD although she lacked her usual symptoms of joint pain, sore throat, chest pain, and rashes (Table 1). She improved with an increased dose of oral prednisone to 30 mg daily. Shortly after that admission, she developed acute on chronic kidney injury with nephrotic range proteinuria and progression to ESRD, believed to be due to progression of CG. Her anakinra dose was reduced to every other day dosing to account for her ESRD.

She was admitted to the hospital in September 2020 for fever, malaise, headache, dyspnea, cough, and diarrhea. On presentation, her temperature was 101.4 F, blood pressure 97/55 mmHg, and heart rate 119 beats per minute. Physical examination revealed an ill-appearing female, normal conjunctiva, unremarkable oropharynx without pharyngeal erythema, sinus tachycardia with no additional heart sounds or murmurs, clear lung fields, benign abdomen without hepatosplenomegaly, unremarkable joint examination without evidence of synovitis, malar rash, chest hyperpigmentation, intact cranial nerves, left arm weakness, intact sensation, and intact reflexes. Anakinra was held due to her fevers and ongoing infectious work-up. Laboratory work-up was significant for leukocytosis, worsening anemia and thrombocytopenia, and CK 4000 U/L (Table 1). CT CAP showed new splenomegaly as well as lymphadenopathy. The patient rapidly deteriorated, resulting in pulseless electrical activity (PEA) arrest and death within a few days of admission. There was concern that she may have progressed to hemophagocytic lymphohistiocytosis (HLH) or macrophage activation syndrome (new cytopenia, splenomegaly, high-grade fevers, profoundly elevated ferritin) but additional supportive laboratory tests and bone marrow biopsy were not obtained. Autopsy revealed bilateral lung consolidation with red hepatization of all five lobes, green–black mucosal discoloration around the gastric fundus, and hepatosplenomegaly with liver weight of 211 g (expected 1500–1800 g) and spleen weight of 414 g (expected 125–195 g). It was concluded that the cause of death was acute respiratory distress syndrome (ARDS) and respiratory failure.

## Methods for literature review

Using the keywords “adult-onset Still’s disease,” “collapsing glomerulopathy,” and “association,” we searched PubMed, Medline, and Cochrane Central to find previously published English language articles reporting on the association between AoSD and CG.

## Results of literature review

There are three published cases in the literature that report an association between AoSD and CG (Table 2).

**Table 2:** Patient characteristics in prior cases reporting an association between AoSD and CG

	Case 1 [4]	Case 2 [5]	Case 3 [6]
Age (years)/Sex/Race	18/Female/African American	32/Male/African American	46/Female/African American
Serum creatinine (mg/dL)	0.76	5.15	2.5
Serum albumin (g/dL)	Unknown	4.1	3.1
Urine protein/creatinine ratio (g/g)	0.19	19.98	5.1
Renal biopsy	Collapsing glomerulopathy	Collapsing glomerulopathy	Collapsing glomerulopathy
Fever	Yes	Yes	Yes
Arthralgias/arthritis	Yes	Yes	Yes
Salmon rash	Yes	Yes	No
Leukocytosis	No	Yes	Yes
Sore throat	No	Unknown	No
Lymphadenopathy	Yes	Yes	Yes
Hepatomegaly/splenomegaly	No	Yes	Yes
AST/ALT/LDH elevation	No	Yes	Yes
ANA/RF serologies	Negative	ANA 1:1280/RF unknown	Negative
Treatment	PO prednisone 1 mg/kg/day and ACE-I	IVIG 0.4 g/kg daily for 5 days and mycophenolate mofetil 1 g BID	Prednisolone 40 mg/day
Clinical improvement	Yes	Yes	Yes

*IVIG* intravenous immunoglobulin, * BID* two times a day

### Case 1

In 2010, Arulkumaran *et al.* reported a case of an 18-year-old African American female who presented with 3 weeks of fever, arthralgias, rash, and lymphadenopathy. Temperature at time of admission was 104 F. Physical examination revealed proximal muscle weakness, submandibular and axillary lymphadenopathy, and knee swelling and joint tenderness. No rash was seen but the patient reported an evanescent rash prior to presentation. Laboratory examination was significant for anemia, ferritin of 1168 ng/mL, and C-reactive protein (CRP) of 220 mg/dL. She had non-nephrotic range proteinuria and underwent a renal biopsy, which was consistent with CG. She met the Yamaguchi criteria for AoSD and was treated with oral prednisolone (1 mg/kg/day) and ACE-I. Within 2 weeks her symptoms had resolved, and inflammatory markers and proteinuria had normalized [5].

### Case 2

In 2004, Bennett *et al.* reported a case of a 32-year-old African American man who presented with 5 months of fever, arthralgias, and rash. Temperature at time of admission was 104 F. Physical examination revealed wrist tenderness, generalized lymphadenopathy, and intermittent maculopapular rash on his limbs. Laboratory examination was significant for leukocytosis, anemia, CRP of 630 mg/dL, and ferritin or 38,691 ng/mL. ANA was initially 1:1280 but then normalized. TTE showed large pericardial effusion and CT abdomen showed hepatomegaly. He met criteria for AoSD and was started on intravenous immunoglobulins (0.4 g/kg/day) for 5 days. Within 3 weeks his symptoms had resolved. He was noted to have nephrotic range proteinuria and underwent a renal biopsy, which showed CG. He was started on mycophenolate mofetil (1 g twice per day) and achieved remission [6].

### Case 3

Kumar *et al.* reported a case of a 46-year-old African American woman who presented with 1 week of fever, arthralgias, and lymphadenopathy. Temperature at time of admission was 103 F. Physical examination revealed knee and ankle tenderness, hepatosplenomegaly, and cervical and axillary lymphadenopathy. Laboratory examination was significant for leukocytosis, anemia, renal insufficiency, nephrotic range proteinuria, lactate dehydrogenase of 1269 U/L, CRP of 550 mg/dL, and ferritin of 4970 ng/mL. She underwent a kidney biopsy, which showed CG. She met the criteria for AoSD and was started on prednisolone (40 mg/day). Within weeks she achieved partial remission and was changed to low-dose maintenance prednisolone. She eventually achieved complete remission [7].

## Discussion

We present a case of a 15-year-old African American female with CG who was diagnosed with AoSD 12 years later. Her CG had been in remission following induction therapy with cyclosporine and prednisone, however following AoSD diagnosis she developed worsening renal function eventually progressing to ESRD. Her AoSD proved to be difficult to control despite various immunosuppressive agents, with frequent flares. During a flare she rapidly deteriorated, resulting in PEA arrest and death. We also present a review of three published cases that report an association between AoSD and CG. In all three cases, CG was diagnosed concurrently or near the diagnosis of AoSD, and remission of both disease processes was achieved with immunosuppression.

AoSD is a diagnosis of exclusion requiring that infection, malignancy, other systemic autoimmune diseases, and drug reactions be ruled out. The Yamaguchi classification criteria was developed to help guide assessment for AoSD. This system requires the presence of five features, with at least two being major criteria. The major criteria include fever of 39 °C for at least 1 week, arthralgias/arthritis for at least 2 weeks, salmon colored rash, and leukocytosis with at least 80% granulocytes. The minor criteria include sore throat, lymphadenopathy, hepatomegaly/splenomegaly, elevations in AST/ALT/LDH, and negative serologies for ANA and RF [8]. The patient’s initial presentation with very high ferritin raised suspicion for AoSD and the patient met the Yamaguchi criteria, given the presence of three major criteria (fevers, arthralgias, and leukocytosis) and three minor criteria (sore throat, lymphadenopathy, and elevations in AST/ALT). The patient had a low titer ANA and SSA positivity, and so SLE was considered, and she was treated with and maintained on hydroxychloroquine. However, through the years of follow-up, she did not develop any other features or specific autoantibodies to suggest SLE. Repeat renal biopsies did not show features of SLE. The fact that her flare-ups were characterized by fevers and high inflammatory markers, including ferritin, and that her best treatment response was to anakinra, was more supportive of a diagnosis of AoSD.

CG features collapse and sclerosis of the entire glomerular tuft rather than segmental injury. Diffuse podocyte injury leads to proliferation, which results in hypertrophy and hyperplasia [3]. It is most often associated with viral infections, medications, genetic causes, or autoimmune/inflammatory diseases (AoSD and SLE) (Table 3). It typically presents with severe nephrotic syndrome and renal insufficiency that can progress rapidly to ESRD. This distinguishes the collapsing variant from other forms of FSGS, which usually have a milder course.

**Table 3:** Causes of collapsing glomerulopathy

**Infections**
Human immunodeficiency virus
Parvovirus B19
Coronavirus
Cytomegalovirus
Epstein–Barr virus
Tuberculosis
Malaria
**Autoimmune**
AoSD
SLE
Mixed connective tissue disease
**Malignancy**
Hemophagocytic lymphohistiocytosis
Multiple myeloma
Acute monoblastic leukemia
**Genetic**
Sickle cell anemia
Mitochondrial cytopathies
Acute myoclonus-renal failure syndrome
**Medications**
Interferons
Pamidronate
Bisphosphonates
Anabolic steroids
Calcineurin inhibitors
**Post-transplantation**
Acute rejection
Thrombotic microangiopathy
Arteriopathy

*AoSD* Adult-onset Still’s disease,* SLE* systemic lupus erythematosus

CG is a rare manifestation of AoSD, with only a few reports noted in the literature. Our case differs from the previously reported cases where CG was diagnosed concurrently or near the diagnosis of AoSD. However, our patient experienced a relapse of her CG at the time of her AoSD diagnosis, which suggests the two diagnoses are not mere coincidence.

The reason for this association is unknown, but may involve interferons (IFNs). IFN-α and IFN-β are primarily produced by virally infected cells and trigger response in neighboring immune cells, while IFN-γ stimulates macrophage activation and major histocompatibility complex (MHC) expression [9]. Although the pathophysiology of AoSD is not yet clear, immunologically mediated inflammation is thought to be involved in the pathogenesis of this disease [10]. Studies have shown that levels of IFN-γ are higher in AoSD than in controls [11]. It is possible that uncontrolled expansion of cytotoxic T lymphocytes and natural killer (NK) cells triggers activation of macrophages and overproduction of cytokine such as IFN-γ. This process has been postulated to be a driver of secondary macrophage activation syndrome in the setting of AoSD [12]. IFN-induced immune responses have been implicated in the kidney damage seen in CG, and this is supported by cases of CG occurring after IFN therapy and certain viral infections [9]. Excessive production of IFNs is also a hallmark of SLE, another autoimmune disease associated with CG. Thus, it is possible IFN may be the common bond between AoSD, SLE, and CG, although more studies are needed to explore this.

## Conclusions

We highlight the rare but important association between CG and AoSD. CG is also seen in patients with SLE and SLE-related disorders. Rheumatologists and nephrologists should be aware of this association, especially when patients with AoSD, SLE, or positive ANA’s develop nephrotic range proteinuria or renal impairment and renal biopsy pathology does not demonstrate lupus nephritis. Additionally, patients with CG should be monitored for signs and symptoms that herald the onset of a new auto-immune/inflammatory disorder, that may even occur years after their initial CG diagnosis, as illustrated by our case.

## Data Availability

Data sharing not applicable to this article as no datasets were generated or analyzed during the current study.
